# Deep learning in systems medicine

**DOI:** 10.1093/bib/bbaa237

**Published:** 2020-11-16

**Authors:** Haiying Wang, Estelle Pujos-Guillot, Blandine Comte, Joao Luis de Miranda, Vojtech Spiwok, Ivan Chorbev, Filippo Castiglione, Paolo Tieri, Steven Watterson, Roisin McAllister, Tiago de Melo Malaquias, Massimiliano Zanin, Taranjit Singh Rai, Huiru Zheng

**Affiliations:** computer science at Ulster University; metabolomic platform dedicated to metabolism studies in nutrition and health in the French National Research Institute for Agriculture, Food and Environment; French National Research Institute for Agriculture, Food and Environment; (ESTG/IPP) and a Researcher (CERENA/IST) in optimization methods and process systems engineering; Molecular Modelling Researcher applying machine learning to accelerate molecular simulations; Faculty for Computer Science and Engineering, University Ss Cyril and Methodius in Skopje, North Macedonia working in the area of eHealth and assistive technologies; Computer Scientist working at the National Research Council of Italy; National Research Council of Italy (CNR) and a lecturer at Sapienza University in Rome, working in the field of network medicine and computational biology; computational biology at Ulster University; Research Associate working in CTRIC, University of Ulster, Derry, and has worked in clinical and academic roles in the fields of molecular diagnostics and biomarker discovery; Research Associate in CTIRC, Derry, UK; Researcher working in the Institute for Cross-Disciplinary Physics and Complex Systems, Spain, with an interest on data analysis and integration using statistical physics techniques; Lecturer in cellular ageing at the Centre for Stratified Medicine. Dr Rai’s research interests are in cellular senescence, which is thought to promote cellular and tissue ageing in disease, and the development of senolytic compounds to restrict this process; Professor of computer sciences at Ulster University

**Keywords:** deep learning (DL), systems medicine (SM), data integration, biomarker discovery, disease classification

## Abstract

Systems medicine (SM) has emerged as a powerful tool for studying the human body at the systems level with the aim of improving our understanding, prevention and treatment of complex diseases. Being able to automatically extract relevant features needed for a given task from high-dimensional, heterogeneous data, deep learning (DL) holds great promise in this endeavour. This review paper addresses the main developments of DL algorithms and a set of general topics where DL is decisive, namely, within the SM landscape. It discusses how DL can be applied to SM with an emphasis on the applications to predictive, preventive and precision medicine. Several key challenges have been highlighted including delivering clinical impact and improving interpretability. We used some prototypical examples to highlight the relevance and significance of the adoption of DL in SM, one of them is involving the creation of a model for personalized Parkinson’s disease. The review offers valuable insights and informs the research in DL and SM.

## Introduction

Systems medicine (SM) has emerged as an interdisciplinary field, which promotes an integrative and holistic approach to studying the human body at the systems level with the aim of improving our understanding, prevention and treatment of complex diseases [1, 2]. The ultimate challenge and vision is a radical shift from a reductionist paradigm to multiscale SM [3] and its real-world validation and patient-relevant application.

As a multiscale, multidisciplinary approach to medicine, SM is characterized by the presence of large amounts of high-dimensional, heterogeneous data ranging from electronic health records (EHRs) to sequencing and multi-omics technologies across levels in tissues and organs [2–4]. It has been suggested that in order to tackle complicated tasks such as the discovery of complex disease patterns with multiple facets from data and realize the full potential of machine learning (ML) in the era of big data, learning models need to go deep and various deep learning (DL) architectures hold great promise in this endeavour [5–7].

Here, we tackle the applications in SM. Other DL reviews are being published under various approaches; some reviews are addressing models and/or methodologies [8–10]; others are focusing either general applications [11] or specific tools (e.g. embedding graphs [12, 13]), or even DL works targeting a certain field (e.g. pharmaceutical research and drug design/discovery [14–16]).

### Introduction to DL

DL is a branch of ML and artificial intelligence (AI) that employs a layered structure of computation to learn data representation with multiple levels of abstraction [17]. The word ‘deep’ in DL implies the number of processing layers through which the raw data are transformed. The ability to progressively build up abstract representation through layer-wise learning and automatically extract relevant features needed for a given task such as image classification and biomarker identification is one of the key advantages of DL [17, 18]. Since the term was first coined and introduced in 1986, DL has brought tremendous performance and remarkable results in numerous domains including image classification [19], signal processing [20] and computational biology and bioinformatics [21, 22], to name a few.

### Multidimensional data in SM

Progress in molecular and medical sciences has led to the accumulation of massive amount of high-dimensional heterogeneous data in SM, which can be structured and unstructured and may come in a variety of fuzzy and noisy forms.

#### Lifestyle data

One major challenge in healthcare systems is to better understand how environmental and lifestyle factors affect health. In particular, the modifiable lifestyle factors are of special interest, especially in non-communicable diseases in which the concept of lifestyle medicine was proposed [23]. In humans, because of the ethical and practical constraints, the capacity of experimentations is limited and therefore research studies are using observational data, without controlled conditions to analyze links between health status and environmental factors [24]. For a long time, conceptual frameworks were proposed to structure categories of health determinants [25]. More recently, the nutritional epidemiology community has successfully implemented data integration platforms [e.g. the Nutritional Phenotype database (www.dbnp.org)] in order to allow joint data analyses at the individual level from multiple nutrition studies. Within the context of the European Nutritional Phenotype Assessment and Data Sharing Initiative (ENPADASI), a metadata was built including the minimal information to connect existing and future studies and increases data sharing [26]. All the efforts will facilitate the integration of data from different sources in order to identify lifestyle determinants at multiple levels in the context of SM.

#### Multi-omics data

The development of omics approaches (e.g. genomics, transcriptomics, proteomics and metabolomics) allowed getting a better understanding of organisms and systems that is the key component of systems biology. However, such analytical platforms generate large and complex data including high analytical variance, intrinsic collinearity and noise presence. Despite large inter-individual variabilities, sample sizes are usually limited by experimental design in comparison to the huge number of collected variables. In this context, dedicated algorithms and tools have been applied to extract the relevant biological knowledge, particularly in the field of ML methods. This challenging task requires minimal reporting guidelines, formats and standards for data management that have been set up in an open science research perspective [27, 28]. At present, large-scale omics data are becoming more available and multi-scale studies requiring multi-omic integration are generalizing because such approaches are of major interest to characterize phenotypes complexity [29]. In this context, DL has emerged as a powerful methodology to both process omic datasets and integrate them for SM [30].

#### Electronic health records

EHRs generally describe an extremely secure healthcare database comprising patient personal information, health-service encounter information, medical histories and diagnoses, management/treatment details, copies of clinical correspondence (such as referral and discharge letters), lab test results, imaging and other specialist investigation data. They are notoriously heterogeneous in their representations and include numerical and categorical values, datetime objects and natural language free-text. The wealth of information contained within free-text sections is especially unstructured and prone to individual clinician writing styles and abbreviations.

Current progress towards integrating EHRs with the demands of data analysis is still at the developmental stage. OpenEHR [31] offers a series of data architectures and standards that have been developed to maximize interoperability and is under consideration for adoption (at least partially) in the future planning of a number of healthcare providers. Fast Healthcare Interoperability Resources proposes data storage standards; but also standards for the accompanying application programming interfaces through which the data can be accessed [32]. Commercial vendors are also beginning to recognize this opportunity, proposing proprietary EHR systems such as Encompass (Epic Systems Inc.) and propriety infrastructure (e.g. Dell EMC Healthcare). Initiatives such as NHS Digital have been created to curate the digital offering of the UK’s National Health Service (NHS) (https://digital.nhs.uk), including the management of secure access to restricted data sets. Academic units have also emerged as organizations that can curate data to support analysis, such as the OpenPrescribing data set that contains historical prescription data [33] and the Connected Health Cities Initiative, which has sought to build an interoperable system with patient engagement on top of existing NHS infrastructure [34].

### DL and SM

Given the numerous applications of classical statistics and data mining in medicine over the past decade [2, 4], one may argue why a new paradigm, i.e. the DL one, is required. To tackle this question, we here consider a prototypical example, involving the creation of a model for personalized Parkinson’s disease (PD) risk estimation. PD is the second most common age-related neurodegenerative disease after Alzheimer’s disease (AD), with an average onset at 55 years, and with symptoms including tremor at rest, rigidity, slowness or absence of voluntary movement, postural instability, and freezing episodes [35, 36]. A key question is still to be answered: can the risk of developing PD be assessed on a personal basis? In other words, given all data that can be collected from a person, can a personalized risk probability be synthesized? As we will here see, the complexity of the problem implies that no simple answer can be manually created, and the nature and structure of the data encoding such answer prevent a solution based on classical data mining algorithms.

One of key components in SM is to use advanced mathematical modelling to integrate multidimensional and multiscale data including both biological and medical data. Recent development and implementation of SM and DL have been possible thanks to the emergence of new tools for multidimensional data generation and integration.

It is now well-known that many cases of PD have a genetic origin, with mutations in the genes encoding the lysosomal enzyme beta-glucocerebrosidase (GBA) and the α-synuclein being associated with, respectively, a 13.6- and 1.23-fold change in the risk of developing PD [37, 38]; strong associations have also been observed for loci GAK-DGKQ, SNCA and the HLA region [39]. Still, a large share of cases is associated with specific behavioural and lifestyle aspects. During the last two decades, it has been shown that exposure (environment, nutrition, etc.) multiplies the risk of PD [40–47]. Most interestingly, various negatively correlated associations are observed with heavy smoking [41–43], alcohol consumption [48], milk and carbohydrates intake [47] and polyunsaturated fat intake [42, 47].

Looking back at the clinical trajectory of each subject, it has been shown that the concomitance of pesticide use, family history of neurologic disease, and depression lead to a probability of developing PD of 92% [40]. Olfaction dysfunctions can predate clinical PD in men by at least 4 years and can thus be a powerful predictor of the disease [49]. Hazard ratios among people with type 2 diabetes, compared with those without it, were around 1.9 [50]. Similarly, prior head injuries with amnesia or loss of consciousness are associated with an increased risk for PD [51], while the use of ibuprofen is associated with a marked decreased risk [52].

Several medical tests can be used to refine PD risk. For instance, PD incidence is higher in the presence of alterations in the human microbiome, due to the relationship between PD, brain and gut [53]. Prodromal stages of PD can manifest as alterations of brain dynamics, as measured by an electro-encephalogram; the risk of developing dementia is then 13 times higher in subjects with low background rhythm frequency [54]. Additionally, a simple blood test can measure the levels of interleukin-6, which are positively correlated with PD [52], and of urate and cholesterol, which are negatively correlated [55, 56]. To conclude, some additional symptoms of early onset of PD have been identified, although being more difficult to measure and, in many cases, only of a subjective nature. These include sleep disturbances, behavioural and emotional dysfunction such as changes of personality, constipation, urinary dysfunction, depressive symptoms and chronic pain in joint and muscle [57, 58].

As clearly appears from even this simple review, creating an automated model for forecasting the risk of developing PD is far from being a trivial task, and one not easily accomplishable with standard statistical and data mining techniques. Large quantities of data have to be collected for each individual; and highly heterogeneous sources processed, such as blood sample analysis, free texts in large collections of EHRs, genetic data and personal interviews. Afterward, all these elements have to be combined into a single model, where relationships may be highly non-linear and may be masked or enhanced by confounding effects. By overpassing these issues, the solution may be at hand thanks to DL.

The purpose of this paper is thus to review DL algorithms and applications in SM, namely: in the Fundamentals of DL models section, the fundamentals of DL models are presented, followed by key contributions of DL to data analytics in medicine; in the DL applications in SM section, applications of DL in SM are revisited; in the Challenges and future trends section, the main challenges and future trends are summarized; and finally, in the Conclusion section, the conclusions and future developments on DL are discussed.

## Fundamentals of DL models

While various DL models have been proposed and developed each exhibiting unique features in its implementation, the core concept behind their success [17, 59] is their ability to perform feature transformation layer-by-layer. A DL network can be considered as a multi-layer perceptron, i.e. a computer model conceived to represent or simulate the ability of the brain to recognize and discriminate, which follows specified rules in the choice of the number of neurons in each layer and in the wiring between layers to enact different representation layers corresponding to conceptual characteristics whose higher layers concepts are defined on the basis of the lower ones.

**Figure 1:**
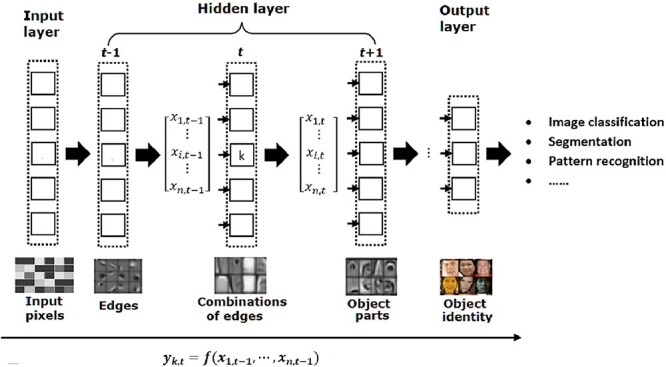
Illustration of the layer-by-layer processing in DL consisting of an input layer, multiple hidden layers and an output layer.

As illustrated in Figure 1, regardless of its architecture, a DL model always works in layers typically consisting of an input layer, multiple hidden layers and an output layer. Each layer contains a certain number of computational units carrying out the transformation of the data received from the previous layer and then passing the results to the next layer as depicted in Equation (1).

(1) \begin{equation*} {y}_{k,t}=f\left({x}_{1,t-1,}\cdots, {x}_{n,t-1}\right), \end{equation*}

where *n* is the number of computational units in the (*t*-1)th layer; and *y_k,t_* and *x_i,t_* stand for the output from the *k*th unit in the *t*th layer and the input from the *i*th unit in the (*t*-1)th layer, respectively.

**Figure 2:**
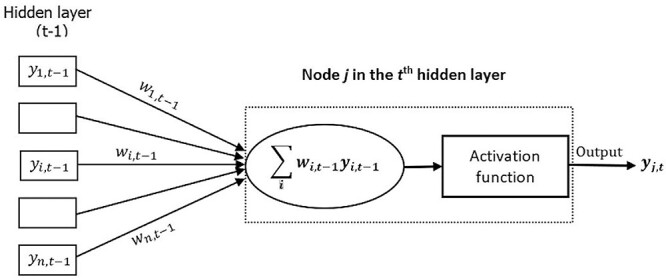
Illustration of a basic building block in a DNN.

With the layer-wise data transformation, deep models are capable of progressively abstracting data representation layer-by-layer, leading to automatic feature engineering from low-level features such as edges to higher, more abstract features like face [17, 60]. In an image classification application in which a raw image is encoded using an array of pixels, the first hidden layer typically detects the presence of various oriented edges at particular locations in the image. The extracted edges are then passed to the next layer which is involved in the detection of some simple shapes such as corners, and subsequent layers would extract more abstract and composite representation such as facial shapes (Figure 1). This represents a huge advantage over traditional shallow ML models in which features need to be extracted and prepared in advance [61, 62]. In particular, the transformation taking place in each layer is performed usually with non-linear functions, generating a set of new features not found in the raw feature space. For example, most current deep models are derived from the artificial neural network and are models using layers of artificial neurons [2]. Each neuron is fully connected to nodes in the previous layer in a manner analogous to biological synaptic connections [63]. To model the behaviour of a biological neuron, a weight representing the strength of each connection to the previous layer is introduced and an activation function is applied on the weighted sum to determine its output to the next level as shown in Figure 2.

Hereinafter, several widely utilized models in DL literature along with their applications in SM are reviewed.

### Recurrent neural networks

**Figure 3:**
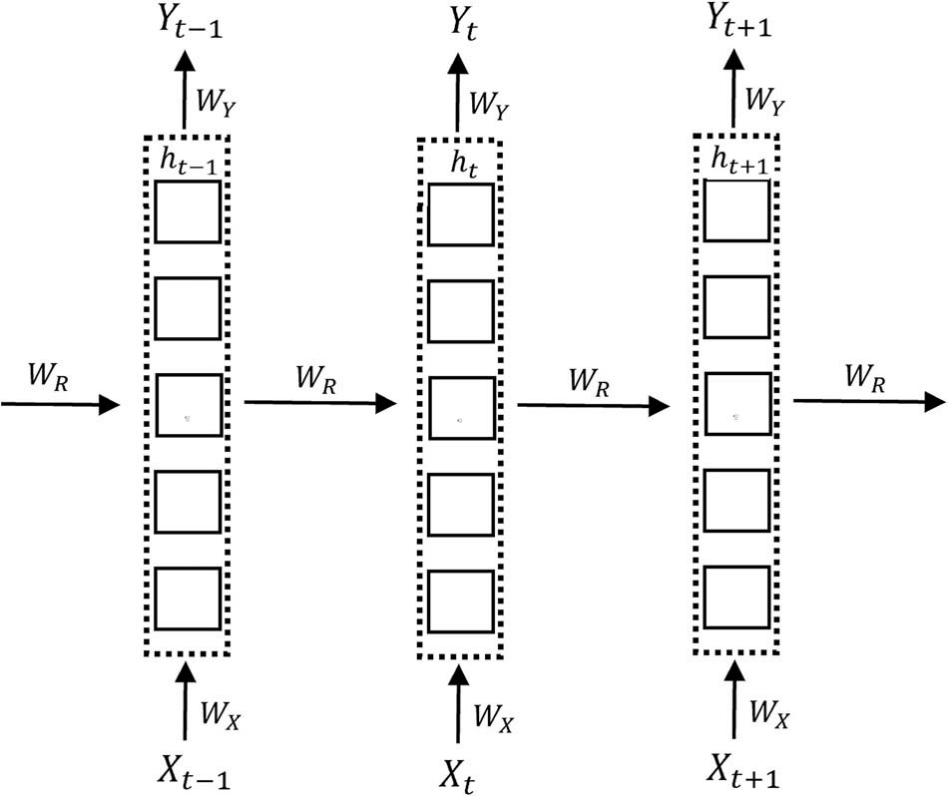
An illustration of the basic structure of an unfolded RNN with an input unit ${X}_t$, a hidden unit ${h}_t$, and an output unit ${Y}_t$ at a sequence index *t*. The weight matrices ${W}_X,{W}_Y\mathrm{and}\ {W}_R$ representing input connection, output connection and recurrent connection, respectively, are shared across the sequence dimension.

A recurrent neural network (RNN) is a DL model designed to make use of sequential information. It has a basic structure with cyclic connection and recurrent units as illustrated in Figure 3, in which the structure is unrolled forward through time.

One of the key features of an RNN is its hidden state, which works as the memory of the network by storing the past information in the hidden units. The state at each time step *t* is estimated based on the previous hidden state and the current input as defined in Equation (2), allowing the network to integrate the states previously learnt through a recurrent approach [22]

(2) \begin{equation*} {h}_t=f\left({W}_R{h}_{t-1}+{W}_X{X}_t\right). \end{equation*}

The structure of an RNN lends itself naturally to the analysis of omics data and biomedical signals which are typically sequential and to modelling temporal dynamic behaviour exhibited by biological processes [30]. For example, a novel RNN approach was introduced to modelling temporal dynamics and dependencies in brain networks observed based on functional magnetic resonance imaging (fMRI) [64]. It has been shown that temporal dynamics can be predicted directly from the recurrent states of the RNN in both task and resting state fMRI. However, due to its recurrent nature, RNN suffers from high computational cost and the problem of gradient vanishing and exploding [65].

### Convolutional neural networks

Inspired by the biological structure and function of the visual cortex, convolutional neural networks (CNNs) have been extensively studied and have become one of the most successful DL models especially in the area of image classification [17]. Pioneering works include the seminal studies published by LeCun *et al.* [66, 67], which established the modern framework of CNNs.

**Figure 4:**
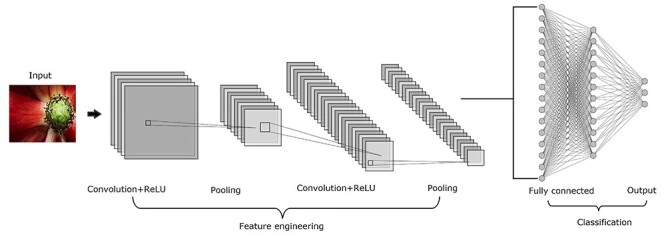
A typical architecture of CNNs which includes four main operations, i.e. convolution, ReLU, pooling and classification with convolutional layers, and pooling layers arranged in an alternating fashion.

A typical CNN architecture includes the following four building blocks as illustrated in Figure 4, in which multiple convolutional and pooling layers are stacked in an alternating fashion in an attempt to learn data with different levels of abstraction.

- Convolution which is central to any CNN models and is used to extract features from data that the spatial arrangement of pixels is preserved. It is a linear operation that involves combining an input matrix with a kernel to produce a feature map whose size is determined by three parameters, i.e. depth (the number of kernels), stride (the number of pixels shifted over the input matrix each time) and padding (the amount of additional pixels added to the edge of an image).
- Rectified linear units (ReLU) which is a non-linear operation applied to convolved feature maps with the purpose of introducing non-linearities in the network.
- Pooling which is a downsampling operation on a rectified feature map aiming to reduce the dimensionality of each map while retaining the most salient features. It involves sliding a 2D filter across a map and summarizing the features selected by the filter. Popular pooling operations include max, average and sum functions with max pooling being the most widely used.
- Classification which is performed based on the output from the convolutional and pooling layers using a fully connected network.

While CNNs require a large amount of annotated data for its proper learning and interpretation of features constructed proves challenging due to its black box nature [68], they have revolutionized the field of computer vision and have been applied to a wide variety of tasks such as medical image segmentation and diagnosis each achieving remarkable performance [69, 70]. Examples include a CNN-based computer-aided detection system developed for detection and classification of lesions in mammograms without any human intervention [71]. The proposed method achieved the state-of-the-art performance on the public INbreast database with AUC = 0.95 and the second place in the Digital Mammography DREAM Challenge with AUC = 0.85.

It is worth noting that while CNNs have been primarily applied to image processing, much effort has been made to apply them to non-image data, which need to be carefully transformed to a well-organized image form [72]. The key component in such applications is to define neighbourhood information ensuring that similar elements are positioned close to each other and dissimilar ones further apart. By converting genomic sequences into 2D image-like data, DeepBind [73] has been successfully applied CNN models to predict the sequence specificities of DNA- and RNA-binding proteins.

### Autoencoders

Autoencoders [74] are a typical DL model designed to learn efficient data representation in an unsupervised fashion. The simplest autoencoder is a feed-forward neural network with an hourglass architecture/shape. The central bottleneck layer separates the neural network into encoding and decoding parts (encoder and decoder). The central layer contains very few neurons; precisely, the number of neurons is equal to the desired dimension of dimensionality-reduced data. The autoencoder is trained to provide a maximum agreement between the signal going into and the signal going out from the autoencoder. A good agreement between the input and output signal reached by the training process implies that high-dimensional data can be dimensionally reduced by an encoder and expanded back by decoder without significant loss of information. Signals in the central bottleneck layer can be used as low-dimensional embeddings of the input data. Autoencoders can be simple (not ‘deep’) neural networks, but they can be deepened by using multiple hidden layers, convolutional and deconvolutional layers, using advanced training methods or other extensions. Autoencoders, however, are data-specific and thus their utility is restricted to applications in which data are considerably similar to the ones used to train the models. In addition, when applied as a data compression algorithm, autoencoders tend to produce a lossy output [75].

Common applications of autoencoders include image denoising [76] and dimension reduction [77]. Since it was introduced in the 1980s [78], a variety of deep autoencoder architectures have been proposed each showing great potential in bioinformatics and SM. Based on a DL strategy, Xu *et al.* [79] introduced a Stacked Sparse Autoencoder to identify distinguishing features of nuclei on high-resolution breast cancer histopathology images. An improved F-measure 84.49% and an average area under Precision-Recall curve 78.83% have been achieved. A three-layer of denoising autoencoder was implemented within a novel framework called ‘deep patient’ used to infer a set of generate features from a large-scale EHR database to facilitate clinical predictive modelling [80].

### Deep generative models

Variational autoencoders [81] and Generative Adversarial Networks [82] belong to a group of deep generative methods. The term generative indicates that these models can generate something. In standard autoencoders, it is possible to point a finger into a random point in the low-dimensional space in the central bottleneck layer. Next, it is possible to decode this signal into the output layer. However, this output signal usually does not have any meaning, especially if the random point lies outside low-dimensional embeddings of the input data. Unlike standard autoencoders, variational autoencoders can generate meaningful output from a random point in the low-dimensional space in the central bottleneck layer. Therefore, they can interpolate and extrapolate high-dimensional training sets. This feature of deep generative models also addresses the fact that dimensionality reduction by standard autoencoders is arbitrary.

As a probabilistic generative model, a deep belief network (DBN) pre-trained using the greedy layer-by-layer learning algorithm was introduced in 2006 [83], which can provide joint probability distributions between input data and labels. It is composed of multiple non-linear layers of latent variables with the connection between top two layers being undirected. One of key advantages exhibited by DBNs is the model that can be pre-trained in a completely unsupervised fashion using a large set of unlabelled data [84]. However, it has been highlighted that DBNs do not take the spatial structure of an image into account, which may significantly affect their performance in some applications [85].

Deep generative models are behind popular applications such as FaceApp (https://www.faceapp.com/), which can modify (extrapolate) an image of a person according to age, visage or gender. Beside such popular applications, deep generative models have a great potential in bioinformatics and SM. Aghdam *et al.* [86] applied DBNs to automatically learn complex mapping from both fMRI and structural magnetic resonance imaging (sMRI) for discrimination of autism spectrum disorders in young children. Abstract high-level features encoded in fMRI and sMRI were extracted and the best performance was achieved with a DBN of depth 3 outperforming the results previously published using Autism Brain Imaging Data Exchange I data.

### Hyperparameters in DL

While each DL model exhibits unique features as summarized in Table 1, there are two types of hyperparameters used in all DL models [87]. The first type is related to model design such as the number of hidden layers in a model, the number of hidden units in a layer and the number of filters in a DNN. The second type is those associated with a learning algorithm including learning rates, activation functions and the number of epochs. The selection of hyperparameters may have a significant impact on the complexity of a DL model and its performance. It has been shown that, in order to realize the full potential of DL, these hyperparameters need to be careful designed [87]. Fortunately, many online DL libraries written in different languages have been made publicly available, which greatly facilitate experimentation. Examples include python-based Keras [88], C++-based Caffe [89] and TensorFlow [90], and Deeplearning4j in Java (https://deeplearning4j.org/).

**Table 1:** A summary of DL models including key benefits, main drawbacks and an example of successful applications

Models	Key benefits	Main drawbacks	Examples of successful applications
RNN	• Learning sequential dependencies in the input	• High computational cost	^a^Modelling both task-related and resting-state fMRI data by capturing its temporal dynamics and dependencies [64]
	• Having internal memory for processing arbitrary sequences	• Gradient vanishing and exploding	
CNN	• Automatic feature extraction	• Black box nature	^b^Detecting and classifying lesions in mammograms [71]
	• Capturing spatial associations	• Requiring enough annotated training data	
Autoencoders	• Learning efficient data representation in an unsupervised fashion	• Data specific	^c^Extraction of high-level features from pixel intensities for the identification of distinguish features of nuclei [79]
	• Dimensionality reduction	• Tend to produce a lossy output	
DBN	• Trained on unlabelled data without supervision	• Computational cost	^d^Automatically learning complex mapping from fMRI and sMRI for discrimination of autism spectrum disorders [86]
	• Exploiting latent feature representation	• Does not account for spatial structure of an image	

^a^An RNN with 100 recurrent hidden units was used. The model was trained with a learning rate of 0.0001 for 500 epochs. Task fMRI data include 28 healthy participants and 24 subjects diagnosed with schizophrenia. Resting state functional MRI data were collected from 55 subjects for 50 min each.

^b^The base CNN used is a 16 layer deep CNN, which was pretrained on 1.2 million images from the ImageNet dataset. The details of the trained model can be found at https://github.com/riblidezso/frcnn_cad

^c^The model used includes the two layer sparse autoencoders each having two hidden layers. There are 400 and 225 hidden units in the first and second hidden layers, respectively. A set of 537 Hematoxylin and Eosin-stained histopathological images were obtained corresponding to 49 lymph node-negative and estrogen receptor-positive breast cancer (LN-, ER+ BC) patients.

^d^Two DBNs having depths of 2 and 3, respectively, were constructed and trained. All the layers have 100 hidden units apart from the top layer in the second DBN, which has 150 hidden units. Pretraining and fine-tuning learning rates of 0.01 were used as hyper parameters for both DBN models. Autism Brain Imaging Data Exchange I and II (ABIDE I and ABIDE II) datasets were used.

One of potentially serious problems when applying DL is overfitting especially when sufficient amount of adequate training is not available. Common techniques to reduce overfitting include the use of regularization [91]. Examples include weight regularization and the dropout approach introduced in 2014 [92]. Parameter sharing in which a set of parameters are shared across layers is another approach for controlling the complexity of a DL model [93].

## Enhancing data analytics in medicine with DL

### Multidimensional and multiscale data analysis and integration

The amount of heterogeneous biological and medical data that are collected and stored on a daily basis is immense and rapidly expanding. However, vast collections of raw data are not in themselves useful. To be meaningful, data must be analyzed and converted into information, or even better, into knowledge. Metabolomics, for example, generates large amounts of complex data reflecting the integration of multilevel regulations. Therefore, modelling approaches adopted in SM are increasingly multiscale [94] and the data processing workflows consist of a multi-step strategy involving various chemometrics and bioinformatics tools [95] in which DL has recently brought new horizons. As an example, DNN has been used for spectral peak classification in the development of several tools that improve data extraction [96, 97]. A DNN-MDA approach has also been shown of interest in determining important variables in complex datasets, in the context of biomarker discovery [98]. Then, DL has shown its powerfulness to explore structural relations between annotated metabolites or proteins, using structural-similarity scoring [99–101]. Finally, Hierarchical multi-label DL was applied to predict enzyme function that can be of great interest for new enzyme design or enzyme-related disease diagnosis [101].

Multidimensional and multiscale data integration is of major interest to model complex biological systems. Using either statistical methods (e.g. correlations), functional analyses or meta-analyses from different studies, they are generally performed to investigate multiscale relations within systems or validity of links between multi datasets across various health status conditions [102]. In this context, DL methodologies were more recently applied to integrate these data. Indeed, such methods have been shown as powerful approaches in their capacity to learn and fit data through representation at multiple levels of abstraction or hidden layer. In fact, Grapov *et al.* [30] reviewed the different DL architectures and their omics applications. One advantage of DL is its capacity to integrate heterogeneous data from different origins, such as clinical data, medical images, molecular multiscale data and even epidemiological ones or parameters from EHR devices.

**Figure 5:**
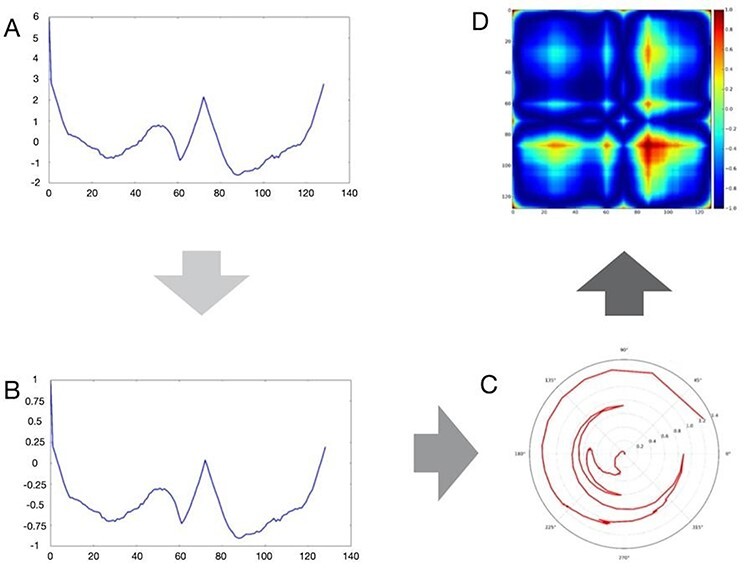
Main steps of the GAF image construction. Starting from an originating time series (A), its ordinate is first scaled to fit the interval [−1, 1] (B), and then translated into polar coordinates (C), and finally to the GAF image (D).

Inspired by recent successes of DL in computer vision and speech recognition, a promising relatively recent methodology has been proposed to encode time series data as images and to classify them using techniques from computer vision, which can be used to apply DL models to analyze various physiological signals such as heart rate, electrocardiogram, electroencephalogram, electromyography and so forth [103, 104]. As illustrated in Figure 5, this method transforms a time series into polar coordinates and then into Gramian Angular Fields (GAF) images [104], i.e. the visual representation of the Gramian matrix, a linear algebra structure used to compute linear independence.

### Biomarker identification

DL has been widely applied in medical image analysis [105] in particular to replace known classifiers and identify new biomarkers [106]. Also, DL algorithms have been used to develop an accurate biomarker of chronological age using eye cornea images [107] and also applied in neuroimaging to identify biomarkers of brain aging using CNNs [108].

**Figure 6:**
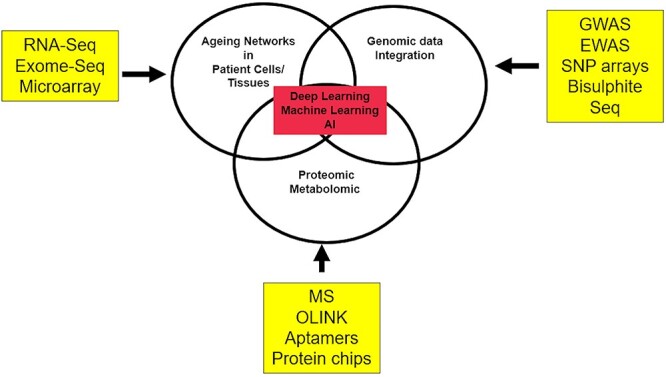
DL for integrative biomarker identification. Abbreviations: Seq-Sequencing, GWAS: Genome wide association.

In addition, DL methodologies can help tease out correct combinations of proteins/genetic signatures that can differentiate between different patient groups from large datasets (Figure 6).

Although the field is still in its infancy, some studies have started to apply the framework. For example, a recent study on atrial fibrillation (AF) integrated genomic, epigenomic and transcriptomic datasets to identify AF-related genes [109]. This study was able to explain the AF variance much better than GWAS alone [109]. Another AF study combined biomarker levels with known clinical risk factors and imaging parameters to differentiate various AF sub-groups [110]. ML algorithms were combined with logistic regression. Results not only confirmed previously published findings such as BNP elevation but also identified FGF-23 as a robust biomarker for AF [110]. AF is an age-associated disease. Traditionally, ageing is viewed as a normal physiological progression towards the death of an organism. However, ageing is the single biggest risk factor for many chronic diseases. One way to address this issue is to radically view ageing as a disease, paving the way to interventions for treating ageing and ageing associated diseases. DL methodologies will be the key to the advancement of these ideas. Indeed, a recent study identified undulating changes in the human ageing process [111]. Using deep mining approaches, the authors suggest these changes as hard coding factors (genomic) to soft coding factors (disease causing) [111]. Similarly, extending such observations to deep multiomics, Ahadi *et al.* [112] showed personal ageing markers change over a short window of 2–3 years. Furthermore, the authors identified what they term ‘ageotypes’ that can reflect ageing, lifestyle and medical history. Ultimately, such discoveries will help in targeting the ageing process [112].

### Disease classification

DL has been extensively applied for disease classification, particularly in cancer research. Tran *et al.* applied DL to identify subtypes from breast cancer gene expression data but also the activity of key transcription factors [101]. Interestingly, this study showed that the deep architecture trained on one dataset could extract the same biological features in other datasets acquired with different technology. DL models also allowed multi-omics integration for identifying survival subgroups of hepatocellular carcinoma [113]. More recently, the DL approach was applied with the same objective to metabolomics data, as an alternative to ML methods. Alakwaa *et al.* [114] showed the higher accuracy of the DL model to predict oestrogen receptor status in breast cancer using a public dataset than when using SVM and RF methods. Moreover, the interpretation of hidden layers allowed identifying eight underlying pathways. In all these publications, DL was undoubtedly of major interest both for an integrative classification of disease subtypes from omics data, but also in terms of interpretation.

The increasing availability of large clinical datasets and medical insurance data with diagnosis and treatment details opened the opportunity to map diseases comorbidities [115]. One of the most interesting papers, which are a motivation behind this work, is the human disease network [116] in which a scalable DL approach was adopted to forecasting disease trajectories over time. The human disease network consists of disorders and diseases linked by the known disorder–gene associations, which offers a platform to explore in a single graph-theoretic framework all known phenotype and disease-gene associations. An RNN containing a memory state was used to integrate medical history into a forecast. Zhang *et al.* [117] proposed a novel CNN for the risk prediction of multiple comorbid diseases from EHRs in which heterogeneous attributes, e.g. diagnoses, procedures and medication, were represented by a graph.

## DL applications in SM

One of the clinical and societal drivers of SM is predictive, preventive, personalized and participatory medicine (P4 medicine) [118]. The vision of P4 medicine has long been advocated by the pioneers of SM [119]. In this section, we describe some successful and promising fields of application for DL in SM with a focus on applications on predictive, preventive and personalized medicine.

### Personalized medicine

Personalized medicine is an overarching approach to medicine where diagnostics, prognostics and prediction of treatment response considers individual-specific factors, rather than those derived from patient populations. Its future role in clinical practice is widely accepted, where it has the potential to streamline and enhance the quality of patient management by improving on the ‘one-size fits all/average patient’ philosophy. The focus is on the individual patient: considering their genotype, phenotype, epigenetics, lifestyle, environmental exposures, etc. With the expanding volume and complexity of medical databases that characterize patients, their diseases and responses, precision medicine is becoming an increasingly viable premise to augment traditional methods [120].

Personized medicine requires a large amount of regularly updated patient-specific data: sociodemographic parameters (e.g. age and gender); medical history; genomics, proteomics and epigenomics; microbiome and infecting pathogens; environmental monitoring, diet and nutrition tracking; and metabolomics, physiological signals and medical imaging [118, 121]. These data are not only of high dimensionality but also unstructured and heterogeneous [122].

Extracting clinical meaning from these data is the first challenge, making robust AI systems crucial. Traditional ML techniques can deal with large amounts of data and can discover hidden patterns and relationships. However, they are ineffective as data dimensionality becomes too large. DL solves this problem as it can deal with a high level of complexity and multi-dimensionality [118]. In medical imaging, it has already demonstrated high potential, powered by the availability of networked architectures and comprehensive labelled datasets [123].

State-of-the-art applications of DL models in SM include tailored treatment plans, drug discovery and development, and accurate disease characteristic identification [118, 123]. For example, Liu *et al.* [124] developed a CNN-based pipeline for MR-based treatment planning in radiation therapy on brain tumor patients, which can produce comparable plans relative to CT-based methods. Suresh *et al.* [125] proposed a CNN model for prediction of clinical intervention within intensive care units. Coupled with patient’s clinical risk factors, an image-based DL framework named Deep Profiler which is capable of individualizing radiation dose, has been developed to deliver personalized radiation therapy to patients [126]. Based on a multimodal DL approach, an integrative framework [127] was developed for the identification of cancer subtypes from multi-platform genomic data, e.g. gene expression, miRNA expression and DNA methylation. By linking to clinical data including patient survival time, time to recurrence and response to drug, it has been demonstrated that the proposed DL-based approach holds promises for understanding subtype-specific transcription programs that controls cancer pathogenesis and tailoring cancer treatment to genetic profiles. To support the development of individualized drug response prediction, Rampasek *et al.* [128] utilized a deep generative model based on variational autoencoders to predict drug response from transcriptomic perturbation signatures. The significant improvement has been achieved demonstrating that the low dimensional latent space derived from the DL model has the potential to encode the essential characteristics of the observed transcriptomic profiles. Thanks to the plethora of the available data and the flexible architecture of DL-based systems, the application of DL in drug discovery for the personalization of therapy has gone beyond compound property and bioactivity prediction [123, 129]. Recent years have seen the rapid development of DL models to address diverse problems in drug discovery such as *de novo* molecular design. Based on a trained deep neural network (DNN), Gómez-Bombarelli *et al.* [130] proposed a novel method to generate chemical structures with desirable properties. Using the deep generative models, Kadurin *et al.* [131] introduced a system which could help develop new molecules with specific anticancer properties.

Accurate disease diagnosis is one of the key milestones for the realization of personalized medicine [123]. Over the past decade, DL-based approaches have achieved remarkable success in diagnosing various diseases thanks to their outstanding performance in biomedical image processing and the ability to incorporate a wide range of individualized features such as genomics, clinical data and lifestyle information. Examples include deep echocardiography representing a DL-based automated diagnosis of cardiac disease [132] and advanced DL models for diagnosis of AD [133] and breast cancer [134]. More recently, a deep representation learning framework namely DeepMicro has been developed for disease prediction based on microbiome data [135], whose role in precision diagnosis and precision medicine has been well recognized [136].

It is anticipated that the incorporation of EHR into predictive modelling could drive personalized medicine. Indeed, several EHR-based DL systems have been developed [137]. Using raw EHR data including free-text notes which formed the patient’s personalized input in temporal order, Rajkomar *et al.* [138] developed DL approaches for the extraction of curated predictor variables from normalized EHR data, and they were capable of accurately producing predictions for a variety of clinical problems (in-hospital mortality, 30-day unplanned readmission, prolonged length of stay and patient’s final discharge diagnoses). In an attempt to improve the characterization of a patient’s clinical phenotype, Rashidian *et al.* [139] applied DL methods to analyze a range of data extracted from EHR (e.g. demographic, laboratory and medication data plus past diagnoses), and to predict International Classification of Disease codes with high accuracy for three test cases (diabetes, acute renal failure and chronic kidney disease). These set of studies contributed to show the importance of DL methods for precision medicine; in addition, they were associated in a good manner with clinical approaches. Nevertheless, it is important to be stated that despite substantial progress has been made in the development of DL-based diagnosis tools, they are mainly used to augment and assist clinicians for relevant tasks [132]. To be adopted for routine use by clinicians, more comprehensive and independent validation is required [126].

### Predictive and preventive medicine

Predictive and preventive medicine is an exciting new approach aiming to predict the probability of a patient developing a disease, thereby enabling either prevention or early diagnosis and treatment of that disease. It has been argued that the future of medicine will move towards predictive and preventive modes [140]. With predictive analytics, both can go hand in hand with the aim of diagnosing disease in its earliest state and preventing its progression further [141].

DL models have been intensively explored in this changeling endeavour. Examples include the recent work by Lu *et al.* [142], which used a CNN to predict long-term mortality from chest radiograph findings and identify persons with an increased risk of mortality at 6 and 12 years, highlighting the prospect of using DL to identify subjects at high risk for adverse outcomes who could benefit from prevention, screening and lifestyle interventions. A DNN was applied to predict multiple cardiovascular risk factors including age, gender, smoking status and systolic blood pressure from fundoscopic eye images that will allow for better cardiovascular risk stratification [143]. Tested in 11 835 UK Biobank participants, the system demonstrates its ability to predict the onset of major adverse cardiovascular events within 5 years.

DL has also been applied to disease staging and outcome prediction. Using chest computed tomography images, Gonzalez *et al.* [144] developed a CNN to identify those individuals with chronic obstructive pulmonary disease, characterize disease severity and predict clinical outcomes including acute respiratory disease events and mortality, which could be used as a powerful tool for risk assessment at a population level.

It has been shown that DL approaches could support the clinician’s decision during each stage of hospitalization, leading to the delivery of better care [145]. Kim *et al.* developed and validated a CNN-based model for real-time prediction of all-cause mortality in critically ill children [146], which may be used for the timely recognition of patients at increased risk of deterioration.

Clinical outcome prediction can be improved by the integration of data contained within patient EHR. The ‘Deep Patient’ prediction system derived a generalizable patient representation [80], using an unsupervised deep feature learning method. It was trained on 700 000 EHRs and used a 3-level noise reduction autoencoder to capture hierarchical regularities within the heterogeneous data. It outperformed raw EHR data in prediction of the development of severe diabetes, schizophrenia and various malignancies.

Beyond the applications for early diagnosis of a disease, DL has shown the potential to improve palliative care. Avati *et al.* [147] applied a fully connected DNN to evaluate all EHR data of all admitted patients and identify those at risk for death within the next 3- to 12-month period. Thus, a proactive approach could be taken to reach out to those who may benefit from palliative care consultation and engage patients and their families in informed decision making.

### DL in action: a case study on PD

As previously introduced in the DL and SM section, PD is a good use case for DL, due to the complexity of the disease and its manifestation, with symptoms usually appearing late and hence preventing an early intervention; and due to the vast array of data that could be used for its study. To conclude this review, we here show some examples of how the previously described DL techniques have been put into action in this disease.

The first natural step towards a better treatment of PD is improving its diagnosis, especially in the case of atypical manifestations, and with the objective of reducing the subjectivity of the process. One of the most characteristic features of PD is that it modifies movement control, and hence initially affects gestures such as writing or drawing spirals. This aspect has been explored by several works, for instance by using CNN [148, 149] and deep Echo State Networks [150], reaching classification scores up to 98% accuracy. Similar classification results have been obtained with features extracted from speech recordings and CNN [151]. The same type of neural networks has further been used to analyze other data, including brain activity [152] and dopamine transporter imaging [153], reaching, respectively, 88.25 and 98.8% accuracy. Beyond the raw classification score, it is important to highlight that these results open the door to the use of data that have previously been disregarded, for being too complex or too subjective in their evaluation, thus expanding the array of tools for diagnosis.

As a second step, DL is expected to trigger a revolution in the way patients are followed, especially in conjunction with the Internet Of Things (IOT) concept. To illustrate, data were recorded with inertial measurement units [154], and it has been shown that the precision in detecting events of bradykinesia, i.e. of the slowness of movement, with DL algorithms was at least 4.6% higher with respect to other state-of-the-art ML techniques. In a similar fashion, DL models have been shown to achieve a 90% precision, as opposed to the 83% of classical classification methods, in the problem of detecting events of freezing of gait. Other examples of the use of wearable sensors, and most notably of motion sensors included in standard smartphones, are presented by several research teams [155–157]. ‘DL will thus allow patients to be followed in their daily life, to analyze data provided by commonly available sensors and to promptly detect adverse episodes and inform the physician about the real course of the disease.

Finally, the ultimate goal of any analysis is to detect ways for slowing down, or ideally stop the progression of the condition. In this sense, a promising line is yielded by drug repurposing. For example, Zeng *et al.* [158] reported a methodology for *in silico* drug repurposing, based on a network deep-learning approach, which integrates known relationships between drugs, diseases, side effects and targets. When results were validated against the ClinicalTrials.gov database, these included previously approved drugs for PD (i.e. methylphenidate and pergolide).

## Challenges and future trends

While massive successes have been achieved in applying DL in SM over the past decade, DL approaches are not without their own limitations [21, 145]. For example, Chen *et al.* argued that traditional ML approaches may produce more interpretable models in some clinical applications [6]. One of the main criticisms against DL is a general lack of interpretability due to its black-box nature [21, 159]. Nevertheless, progress has been made in improving the interpretability of DL in healthcare [114, 160, 161]. For example, by highlighting patient trajectories that maximally activate CNN predictions, Suresh *et al.* [125] improved the interpretability when applying the CNN to predict clinical intervention.

In contrast to traditional ML models, DNNs contain far more learning parameters that need to be determined. One may argue that the large number of hyperparameters shared by DNNs makes them an appropriate model of the brain [162]. It, however, poses two great challenges [21]. Most DL algorithms have assumed sufficient and balanced training data, which may not be the case in some SM applications. Chen *et al.* compared 5 ML methods with 2 DL models using 5 clinical datasets and found that conventional ML methods generated better performance when compared with the DL alternatives in most of cases when training data are relatively small. For instance, when applied to the prediction of time to first treatment or patients diagnosed with chronic lymphocytic leukemia, the highest AUC value (0.924) was obtained by Random Forest and the DL model only achieved an AUC of 0.802. In addition, having many parameters tends to make a model adapt to the data too much, though the risk of overfitting could be reduced through various regularization approaches such as dropout [17, 92]. To address these challenges, Zhou and Feng [5, 59] proposed a new DL method named Deep Forest (gcForest) which is realized by non-differentiable units. It has been shown that gcForest has much fewer parameters in comparison to DNN and can work well even when there are only small-scale data available. A multi-weighted gcForest has been proposed and developed as a staging model of lung adenocarcinoma based on multi-modal genetic data which could be used for the diagnosis and personalized treatment of lung cancer [163].

It has been suggested that the promise of DL maybe ‘overhyped’ [164]. They predicted that DL expectations are inflated and that this bubble may burst. This is becoming a subject of heated debated. Abrol *et al.* [165] argued that DL models have the potential to substantially improve compared with traditional ML techniques if implemented following the prevalent DL practices in particular when applied to the applications with the presence of non-linearities in data such as brain imaging data. Based on the analysis of 12 314 sMRI images taken from the UK Biobank repository, they demonstrated that DL approaches significantly surpassed ML models and consistently achieved better performance with an increase of sample size. Song *et al.* [166] reported a CNN-based AI assistance system deployed at the Chinese PLA General Hospital for gastric cancer detection. It underwent a 3-month trial run with the daily gastric dataset and the stable performance with AUC above 0.98 was achieved across timeline. To prove its clinical utility, the system was tested using the data collected from other hospitals, i.e. Peking Union Medical College Hospital and Cancer Hospital, Chinese Academy of Medical Sciences. Consistent performance was achieved, demonstrating the feasibility and benefits of using DL-based histopathological assistance systems in routine clinical practice scenarios. However, a recent investigation published by Nagendran *et al.* [167] highlights that while DL-based approaches have the potential to improve clinical outcomes, claims of DL outperforming clinicians may be exaggerated which could pose a risk for patient safety. To avoid hype and protect patients, it has been suggested to enhance clinical relevance and develop a rigorous evidence base, which are transparently reported in DL studies.

Delivering clinical impact is one of the key challenges for applying DL in SM [159]. While several clinically applicable DL systems have been developed [166, 168, 169], it has been argued that translating advanced DL technologies from research to clinical practice requires careful consideration and system design [159]. Robust clinical evaluation and using clinically applicable metrics that go beyond traditional assessment from a technical perspective are essential.

The challenges and future trends for DL and SM shall consider the available EU funding and networking opportunities and initiatives. Application of DL to SM has sparked many collaborative projects in industry and academia. For example, the interaction of young researchers with other scientific disciplines is ongoing on the crossroads of DL and multiscale computing within the COST Action *OpenMultiMed* (CA15120, https://www.cost.eu/). The role played by DL in data analytics in biomedicine has been highlighted in the report [170] recently released by the Innovative Medicine Initiative (IMI), which includes the use of AI to select the best cancer treatment in its last Calls for proposals under the IMI2 programme (https://www.imi.europa.eu/news-events/press-releases/imi-launches-final-imi2-calls-proposals). One of targeted impacts to be delivered by the next EU research and innovation framework program (2021–2027), i.e. Horizon Europe, is to unlock the full potential of new tools, technologies and digital solutions for a healthy society [171]. It is envisaged that elements of new data analytics such as DL-base approaches would be found in the forthcoming programs.

## Conclusions

Recent years have seen a growing interest in the adoption of DL models across various branches of SM research. This review paper addressed the main developments of DL algorithms and a set of general topics where DL is decisive; namely, within the SM landscape. It informs about the associated applications in SM with an emphasis on the applications to predictive, preventive and precision medicine. The key advantages and limitations were presented too, while challenges and future trends for the DL research are discussed.

While DL models have achieved outstanding performance in SM recently, translating the research into clinically applicable systems and delivering clinical impact represent a big challenging task. One of the key requirements is a robust clinical evaluation that needs to be based on the metrics taking the quality of care and patient outcomes into consideration [159]. Other factors to be considered include further improvement of the interpretability of DL predictions and transformation of DL away from its current black box model, through, for example, the visualization of hidden layers and the enhancement of human-algorithm interactions [21, 159].

It has been highlighted that participatory medicine is becoming a driving force for revolutionizing healthcare [172]. The evolution toward participatory medicine can be boosted by the application of the IOT involving the use of DL [173]. Examples include detection of AF using a commercially available smartwatch coupled with a DNN [174] and CNN-based gesture pattern recognition [175]. Still, the application of DL to participatory medicine is at its early stage and its impact on patient care deserves further investigation [176].

DL is becoming an important computational tool to decipher the complexity of diseases and playing a significant role in analyzing heterogeneous data generated in SM [119]. Nevertheless, it is important to mention that DL is not a silver bullet [21] and some claims of DL superiority may constitute a hype which deserves further scrutiny [167]. Translating DL technologies into a clinically validated system is still a challenging task, but significant progress has been made. The review presented offers valuable insights and informs the research in DL and SM.

**Key Points**

- As a multiscale, multidisciplinary approach to medicine, systems medicine (SM) is characterized by the presence of large amounts of high-dimensional, heterogeneous data.
- In order to tackle complicated tasks such as the discovery of complex disease patterns with multiple facets from data and realize the full potential of machine learning in the era of big data, learning models need to go deep and various deep learning (DL) architectures hold great promise in this endeavour.
- This review paper addresses the main developments of DL algorithms and a set of general topics where DL is decisive; namely, within the SM landscape. It informed about the associated applications in SM with an emphasis on the applications to predictive, preventive and precision medicine.
- Several key challenges have been highlighted including delivering clinical impact and improving interpretability.

## Notes

**Haiying Wang** is a Reader in computer science at Ulster University. His research interests lie broadly within the areas of machine learning, integrative data analysis and network-based approaches to systems biology.

**Estelle Pujos-Guillot** is the Head of a metabolomic platform dedicated to metabolism studies in nutrition and health in the French National Research Institute for Agriculture, Food and Environment. Her research interests concern metabolomics and data mining to increase knowledge extraction from high-throughput data.

**Blandine Comte** is a Research Director working at the French National Research Institute for Agriculture, Food and Environment. Her research interests are focused on understanding the mechanisms by which nutrition contributes to the development or the prevention of non-communicable chronic diseases.

**Joao Luis de Miranda** is a Professor (ESTG/IPP) and a Researcher (CERENA/IST) in optimization methods and process systems engineering. He is also addressing international cooperation in multidisciplinary frameworks, and serving in several boards/committees at the national/European level.

**Vojtech Spiwok** is a Molecular Modelling Researcher applying machine learning to accelerate molecular simulations.

**Ivan Chorbev** is a Professor at the Faculty for Computer Science and Engineering, University Ss Cyril and Methodius in Skopje, North Macedonia working in the area of eHealth and assistive technologies.

**Filippo Castiglione** is a Computer Scientist working at the National Research Council of Italy. His research interests range from the modelling of biological systems, to machine learning and high-performance computing.

**Paolo Tieri** is a Researcher at the National Research Council of Italy (CNR) and a lecturer at Sapienza University in Rome, working in the field of network medicine and computational biology.

**Steven Watterson** is a Lecturer in computational biology at Ulster University. His research interests lie in systems biology and machine learning in the life sciences.

**Roisin McAllister** is a Research Associate working in CTRIC, University of Ulster, Derry, and has worked in clinical and academic roles in the fields of molecular diagnostics and biomarker discovery.

**Tiago Malaquias** works as a Research Associate in CTIRC, Derry, UK. His research has been focused on the identification of proteomic biomarkers using machine learning algorithms.

**Massimiliano Zanin** is a Researcher working in the Institute for Cross-Disciplinary Physics and Complex Systems, Spain, with an interest on data analysis and integration using statistical physics techniques.

**Taranjit Singh Rai** is a Lecturer in cellular ageing at the Centre for Stratified Medicine. Dr Rai’s research interests are in cellular senescence, which is thought to promote cellular and tissue ageing in disease, and the development of senolytic compounds to restrict this process.

**Huiru Zheng** is a Professor of computer sciences at Ulster University. Within her broad research in machine learning and systems medicine, Prof. Zheng has particular research interest and expertise in systems biology and assistive technology to support independent living.
